# Case Report: Integrative management of refractory fibromyalgia with pulsed electromagnetic fields and ozone therapy: a case series

**DOI:** 10.3389/fmed.2026.1763506

**Published:** 2026-04-15

**Authors:** Jesús Antonio Lara-Reyes, Gonzalo Emiliano Aranda-Abreu, Xamanek Cortijo-Palacios, Arnoldo Aquino-Gálvez, Manuel Castillejos-López, Erick Gerardo Ruiz-Moya, Fausto Rojas-Durán

**Affiliations:** 1 Instituto de Investigaciones Cerebrales, Universidad Veracruzana, Xalapa, Veracruz, Mexico; 2 Centro de EcoAlfabetización y Dialogo de Saberes, Universidad Veracruzana, Xalapa, Veracruz, Mexico; 3 Instituto Nacional de Enfermedades Respiratorias, Ciudad de México, Mexico; 4 Centro más Longevidad, Xalapa, Veracruz, Mexico

**Keywords:** case report, fibromyalgia, integrative medicine, ozone therapy, pulsed electromagnetic field therapy, refractory pain

## Abstract

Fibromyalgia is a complex, heterogeneous and often disabling syndrome that seriously affects the quality of life of patients. It is characterized by chronic widespread musculoskeletal pain, fatigue, sleep disturbances, and cognitive impairment. Its pathophysiology remains poorly understood, and conventional treatments often offer limited efficacy while carrying significant side effects, highlighting the urgent need for alternative therapeutics strategies. We report a retrospective case series evaluating a novel protocol that combines low frequency Pulsed Electromagnetic Field (PEMF) therapy, medical ozone insufflation, and targeted nutritional supplementation in three patients with refractory fibromyalgia who had previously shown inadequate response to conventional treatments. Following the intervention, patients experienced substantial pain relief and significant improvement in quality of life, which were sustained at the 30-day follow-up assessment. These findings suggest that the combination of PEMF and ozone therapy may induce durable physiological benefits, presenting a promising complementary strategy for managing refractory fibromyalgia.

## 1 Introduction

Fibromyalgia (FM) is a complex, heterogeneous, and often disabling syndrome whose pathophysiology is not fully understood, involving central sensitization, neuroendocrine dysfunction, and inflammatory pathways (1). It is primarily characterized by chronic widespread musculoskeletal pain, fatigue, sleep disturbances, cognitive impairment (“fibro fog”), and a range of other somatic symptoms, significantly impacting patients’ quality of life (2). With a prevalence of 2–3% and a higher incidence in women, FM management remains a significant clinical challenge due to the limited efficacy and notable side effects of many conventional pharmacological treatments (3, 4). Although drugs like duloxetine, milnacipran, pregabalin, and amitriptyline offer benefits for fibromyalgia, their efficacy is often limited in refractory cases, and adverse effects frequently impair adherence and quality of life (5). High discontinuation rates—up to 48% for pregabalin and 42% for duloxetine within 12 months—highlight issues of tolerability and insufficient effectiveness (6). Amitriptyline is linked to dropout due to side effects like dry mouth and sedation, while providing satisfactory pain relief for only a minority (7–9). Milnacipran may show reduced analgesia at certain doses (10). These limitations emphasize the need for complementary strategies (5).

“Pulsed electromagnetic field (PEMF) therapy has emerged as a promising non-invasive modality. Low-frequency PEMF has been shown to reduce pain and improve functional status and quality of life in FM patients, potentially by modulating neuronal activity, reducing inflammation, and improving microcirculation (11, 12). Similarly, ozone therapy has been explored for its potential benefits in various musculoskeletal and chronic pain conditions, including fibromyalgia. However, it is important to acknowledge that large-scale randomized controlled trials specifically validating rectal ozone therapy for fibromyalgia remain limited. Some studies (13, 14) have indicated possible therapeutic effects and a favorable safety profile for ozone therapy in general, suggesting that rectal insufflation could be a less invasive alternative. Nonetheless, these findings underscore the need for more rigorous research through controlled trials with adequate sample sizes to definitively establish its efficacy. Its proposed mechanisms may involve the modulation of the immune response, reduction of oxidative stress, and improvement of oxygen metabolism, leading to analgesic and anti-inflammatory effects (15, 16). Our case series aims to contribute to this emerging evidence by evaluating an integrative protocol that includes rectal ozone insufflation in refractory fibromyalgia patients.

While therapeutic exercise remains a cornerstone of fibromyalgia management as recommended by current guidelines (17, 18) the patients included in this study had already followed such prescribed programs within their conventional treatment regimens without achieving sufficient symptomatic improvement. Therefore, this case series was designed to evaluate an integrative, adjunctive protocol combining PEMF therapy and medical ozone insufflation, alongside nutritional support, in this specific subgroup of patients with treatment-refractory symptoms. The aim was to explore complementary therapeutic strategies that could offer additional benefit to those who continue to experience significant symptom burden despite adherence to guideline recommended nonpharmacological approaches, including exercise.

Given the multifactorial nature of FM, we set out to determine if a combination of therapies that were applied targeting different pathways generated better outcomes. Therefore, this case series describes the novel application of a combined protocol using PEMF and ozone therapy, alongside nutritional supplementation, in three patients with long-standing, treatment-refractory FM, evaluating outcomes with validated tools and the Reliable Change Index to objectively demonstrate improvement. Clinical data were collected retrospectively from medical records and from the outcome measures documented during the treatment period.

## 2 Case description

### 2.1 Patient information

Three consecutive female patients with a confirmed diagnosis of FM according to the American College of Rheumatology (ACR) criteria were included (19). All three patients had a long history of the disease exceeding 10 years and had been followed by multiple specialists across different institutions prior to referral to our center. Due to the extended disease duration and fragmented medical records, complete documentation of all prior treatments was not available. However, at the time of admission, all patients were under active follow-up with their respective physicians and were receiving conventional pharmacological treatments—including analgesics, antidepressants, and anticonvulsants—without achieving adequate symptom relief. This persistent symptom burden despite ongoing standard therapy, in combination with symptom chronicity defined their condition as refractory FM. While none of the patients reported a significant family history of rheumatic or autoimmune disorders, relevant psychosocial stressors were identified, including work-related stress in Case 3 and notable familial stress in Case 2, which are recognized as potential exacerbating factors in FM.

All patients were referred to the treatment center with a pre-existing diagnosis of fibromyalgia established by other specialists. Upon admission, we conducted a systematic diagnostic confirmation process following ACR criteria (19). Given the complex presentations, which included comorbid mood and gastrointestinal disorders, differential diagnoses such as systemic lupus erythematosus, rheumatoid arthritis, and hypothyroidism were systematically ruled out through clinical history, physical examination, and available laboratory records. The final diagnosis of refractory fibromyalgia was established based on: symptom chronicity (>3 months), fulfillment of ACR criteria with elevated Widespread Pain Index (WPI) and Symptom Severity (SS) scores, exclusion of other explanatory pathologies, and documented inadequate response to multiple lines of conventional therapy. This structured approach ensured diagnostic rigor and appropriate patient selection for the present case series.

#### 2.1.1 Case 1

A 63-year-old female psychologist with a 20-year history of FM, accompanied by comorbid depression, widespread pain, decreased appetite, and food intolerances. Relevant physical examination findings in this patient at baseline revealed widespread tenderness upon light palpation, aligning with the classic FM tender point distribution, particularly in the cervical, trapezius, and paraspinal regions. Notable muscle stiffness was observed in the paraspinal and trapezius muscles, with reduced cervical range of motion. No focal neurological deficits were detected. Notable muscle stiffness was observed in the paraspinal and trapezius regions. Her pharmacological regimen at the time of clinical evaluation includes clonazepam, venlafaxine, pregabalin and celecoxib.

#### 2.1.2 Case 2

A 52-year-old female with a 12-year history of FM, anxiety, depression, frequent dizziness, and gastrointestinal issues. Physical examination at admission identified generalized allodynia and hyperalgesia upon palpation. The patient presented with significant stiffness in the cervical and lumbar spine, with active range of motion limited by pain. Although no focal neurological deficits were detected, frequent dizziness was reported. Muscle tone was increased in the paraspinal and shoulder girdle muscles. Her medication regimen included Clonazepam, Venlafaxine, Pregabalin, and Celecoxib.

#### 2.1.3 Case 3

A 62-year-old female lawyer with a 14-year history of FM, insomnia, anxiety, headaches, and frequent painful urination whose previous urological evaluation ruled out urinary tract infection, interstitial cystitis, and structural abnormalities. Initial clinical assessment of this patient demonstrated diffuse musculoskeletal tenderness, particularly in the axial skeleton and large proximal joints, including the cervical spine, shoulders, and hips. The examination was also significant for muscle tension in the occipital area, potentially associated with her reported headaches. Pelvic floor muscle hypertonicity was identified on examination, consistent with her symptoms of frequent painful urination after urological pathology was excluded. No focal motor or sensory deficits were noted. These objective physical findings provided a clinical basis for her symptomatic presentation. Her medication regimen included pregabalin.

These findings are congruent with the central sensitization phenotype typical of FM and provide a clinical correlation to the elevated WPI scores recorded at the initial assessment in all three patients (Table 1).

**Table 1:** Baseline (BL) to end-of-treatment (session 21) changes in FIQ, WPI, and SS-Score scores with corresponding reliable change indices (RCI).

Measure	Case 1		Case 2		Case 3	
	BL→S21	RCI	BL→S21	RCI	BL→S21	RCI
FIQ (%)	75→21	−7.98*	59→40	−2.81*	65→4	−9.01*
WPI	12→4	−5.84*	18→13	−3.65*	9→0	−6.57*
SS-Score	8→4	−4.08*	9→8	−1.02*	8→0	−8.16*

All patients showed statistically reliable improvement (RCI| > ±1.96) in FIQ and WPI. SS-Score improvements were reliable in Cases 1 and 3. Asterisks (*) denote statistically reliable change (*p* < 0.05), indicating treatment effects unlikely due to measurement error or chance variation.

### 2.2 Therapeutic intervention

The selection of this integrative protocol was based on the complementary mechanisms of action of PEMF and ozone therapy. PEMF was chosen for its documented effects on neuronal modulation, anti-inflammatory pathways, and microcirculation (11, 12), while ozone therapy was included for its immunomodulatory, antioxidant, and analgesic properties (15, 16). The combination was hypothesized to produce synergistic effects addressing the multifactorial pathophysiology of fibromyalgia. All patients received the same integrative protocol:

*PEMF Therapy:* The PEMF device (EIMA, Magnetic Inductive Stabilizer) was applied systemically. During each session, the patient remained seated with the forearm placed inside the applicator for a duration of 45 min. Using a frequency of 60 Hz, a semi-sinusoidal wave form, with a duty cycle of 12% in a repetitive single-pulse mode (true pulsed mode). The treatment was administered using a magnetic flux of 0.1968 Tesla (equivalent to 4.92 mT/kg), twice weekly (see Supplementary Figure 4).
*Ozone Therapy:* Rectal insufflation of 100–200 mL of medical-grade ozone at a concentration of 28 μg/mL, administered twice weekly. The ozone concentration was fixed at 28 μg/mL for all patients throughout the treatment, following protocols established by the Scientific Society for Oxygen-Ozone Therapy (13, 20). The volume of the gas mixture (ozone/oxygen) was individually titrated based on patient tolerability and colonic capacity, starting at 100 mL and gradually increasing to a maximum of 200 mL by the end of the treatment course, as tolerated.
*Supplementation:* Daily Omega-3 fatty acids and Vitamin D3.

The total treatment course comprised 21 sessions. Existing stable medication regimens were not altered during the active treatment phase for the purpose of this observation, though patient-reported changes were noted.

### 2.3 Diagnostic assessment, follow-up, and outcomes

Clinical assessment was performed using the Widespread Pain Index (WPI), Symptom Severity Score (SS-Score), and the Fibromyalgia Impact Questionnaire (FIQ) (19, 21). The WPI and SS-Score were derived from structured clinical interviews conducted by the treating physician at each timepoint (clinician-assessed outcomes). The FIQ was self-administered by patients, reflecting their subjective perception of functional impact and quality of life (patient-assessed outcomes). Evaluations were conducted retrospectively at baseline, after treatment sessions 7, 14, and 21, and at a 30-day post-treatment follow-up. The Reliable Change Index (RCI) was calculated for scores at session 21 compared to baseline to determine statistically reliable change (RCI > ±1.96) (Table 1).

The results demonstrated marked and sustained improvements across all key symptom domains. All three patients exhibited statistically reliable reductions in the WPI (RCI: −5.84, −3.65, −6.57) and FIQ (RCI: −7.98, −2.81, −9.01), confirming significant alleviation of pain and enhancement of quality of life (Supplementary Figures 1, 3). Reliable improvement in the SS-Score was observed in two of the three patients (RCI: −4.08, −8.16); the third patient, despite a stressful life event during treatment, still showed a positive trend (Supplementary Figure 2). Crucially, a reduced reliance on conventional pharmaceuticals such as clonazepam, pregabalin, venlafaxine and celecoxib were reported in all patients, underscoring the protocol’s potential to mitigate polypharmacy. The therapeutic benefits were not only significant immediately post-treatment but were also maintained at the 30-day follow-up, suggesting the induction of durable physiological modifications. The intervention was well-tolerated, with no reports of significant adverse effects. All three patients completed the full 21-session treatment course, demonstrating 100% adherence to the protocol. No sessions were missed or rescheduled. Minor, transient sensations of warmth at the PEMF applicator site were reported by two patients during the initial sessions, which resolved spontaneously and did not require intervention or session interruption. No serious adverse events occurred during the study period. Specifically, no patients experienced pain exacerbation, skin reactions, gastrointestinal disturbances related to ozone therapy, or systemic adverse effects. No unanticipated events were recorded.

Data visualization and statistical plotting were performed using Plotly Studio (Plotly Technologies Inc., Montreal, Canada) and SPSS Statistics 30.0.0 (International Business Machines Corp., New York, United States).

**Figure 1:**
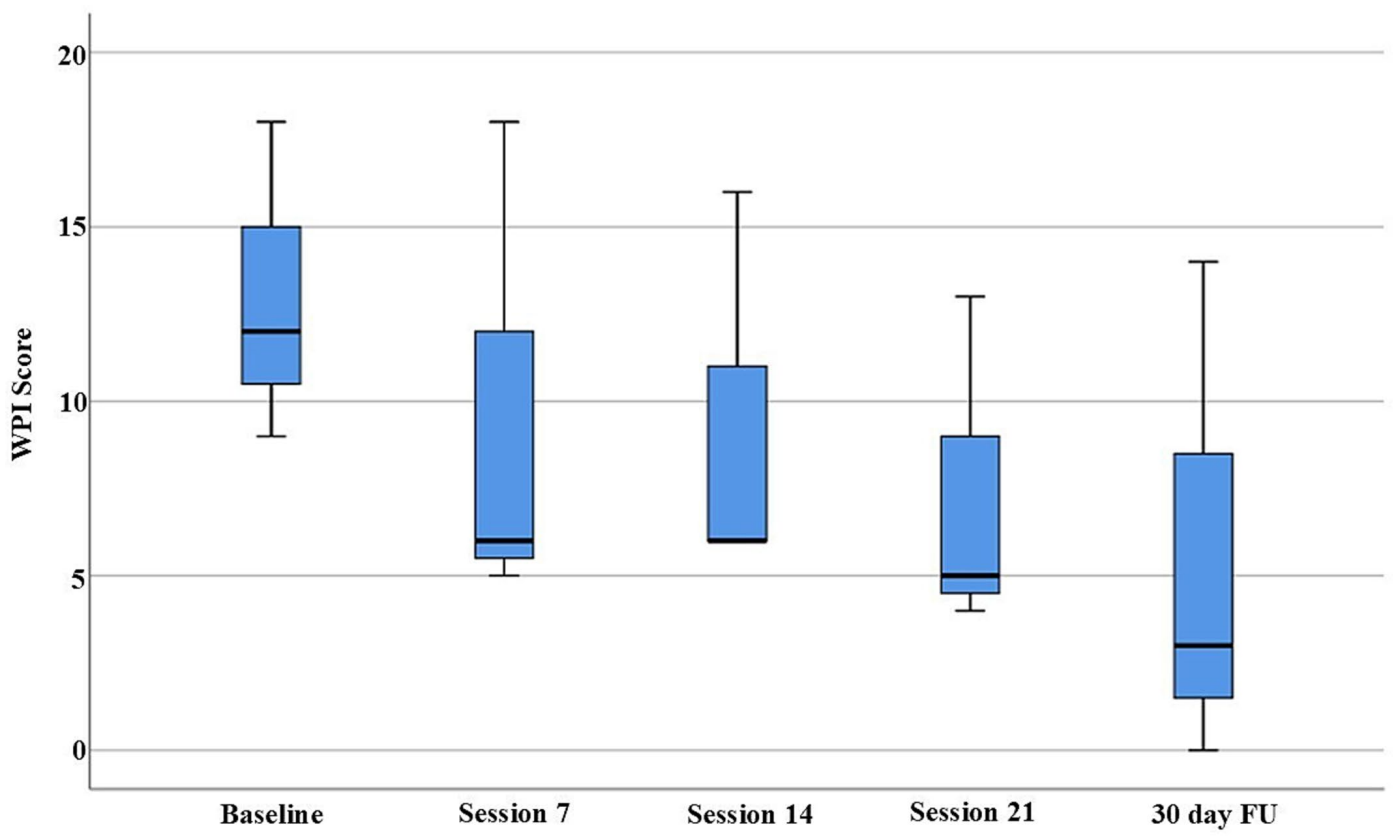
WPI scores exhibited a consistent reduction in pain distribution across all three patients following the integrative PEMF and ozone therapy. The largest decrease occurred between baseline and session 7, with continued improvement observed through sessions 14 and 21. Statistically reliable improvements were evident at the individual level (*y*-axis, 0 to 20), and the longitudinal analysis confirmed a significant group-level change across treatment time points (Friedman test, *p* = 0.030). All patients maintained these clinical gains at the 30-day follow-up (30-day FU), supporting the durability of the therapeutic effects beyond the active intervention period.

**Figure 2:**
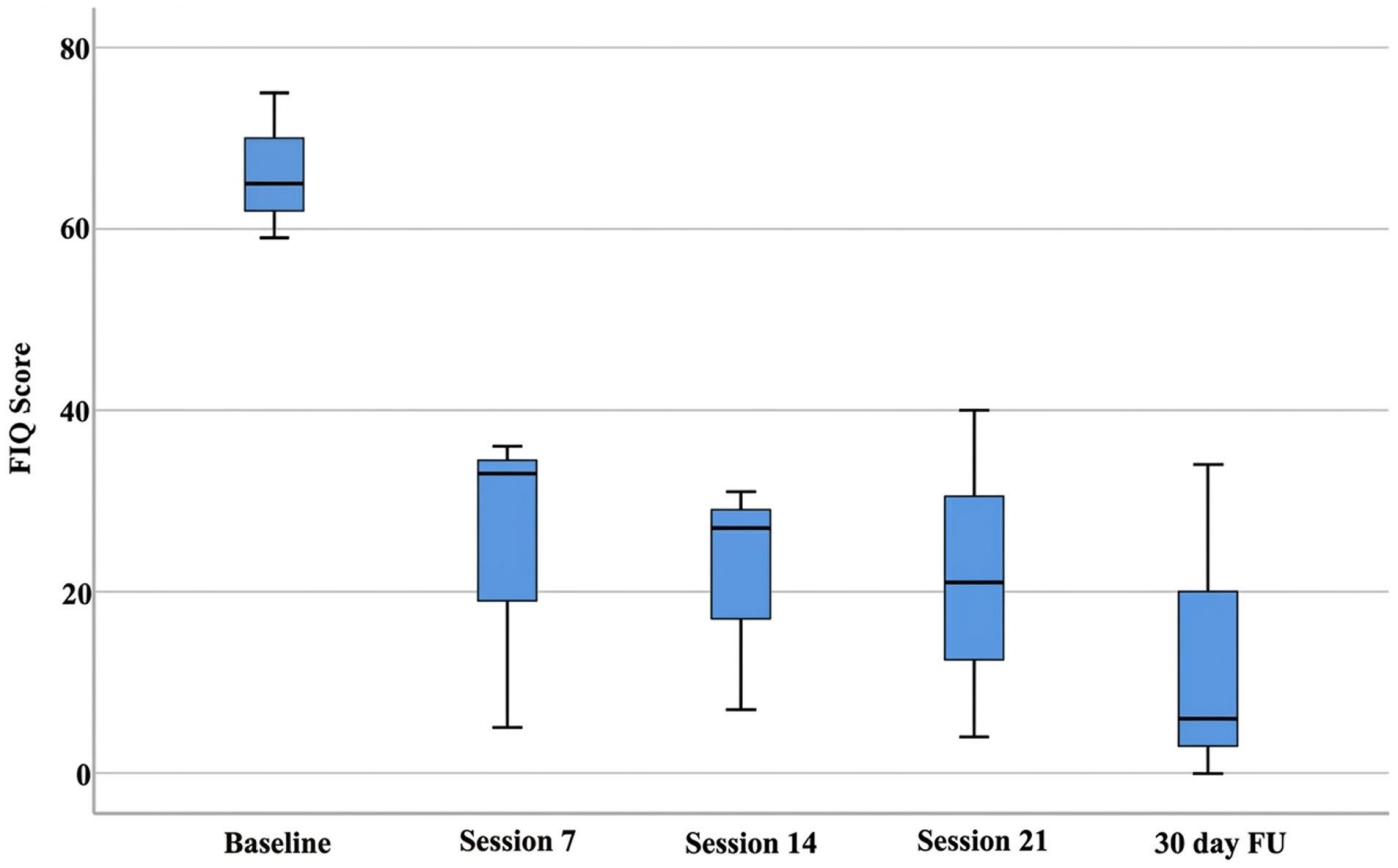
FIQ scores indicate a significant improvement in quality of life and functional impact. This box plot displays score distributions (*y*-axis, 0 to 80) across five time points: baseline, session 7, session 14, session 21, and 30-day FU (*x*-axis). The plot reflects consistent individual-level treatment efficacy, and the longitudinal analysis revealed a significant group-level improvement (Friedman test, *p* = 0.044). These benefits were maintained at the 30-day follow-up, indicating sustained clinical gains beyond the active treatment phase.

**Figure 3:**
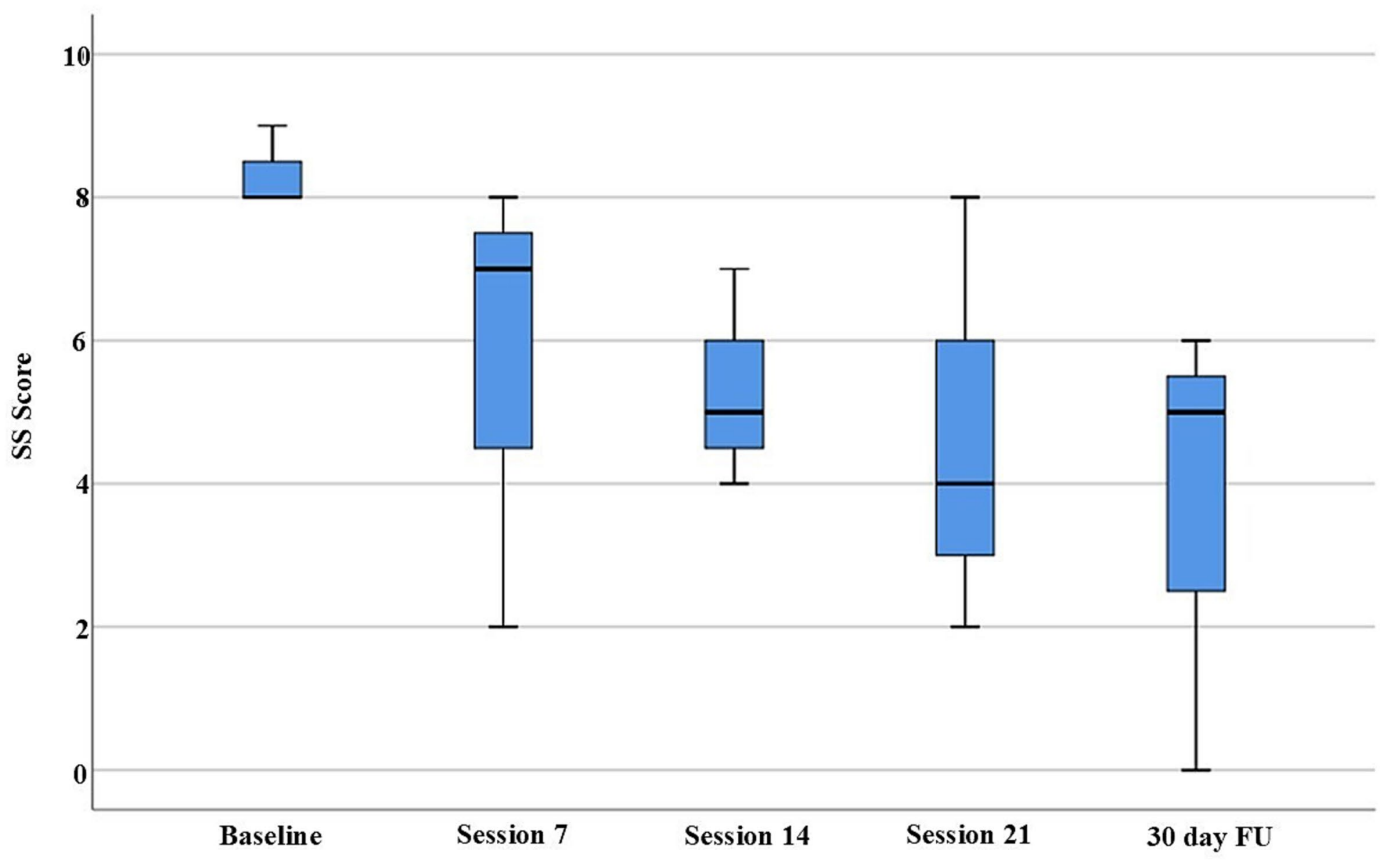
The box plot illustrates SS scores across five time points (baseline, session 7, session 14, session 21, and 30-day FU). Patients demonstrated stable maintenance of symptom improvement at the 30-day follow-up. Although the longitudinal analysis suggested a positive trend (Friedman test, *p* = 0.108), it did not reach statistical significance, likely due to the limited sample size.

Beyond individual reliable change indices, group-level Friedman tests, were employed to assess the consistency and significance of observed changes within the limitations of the small sample size. Friedman tests were conducted to assess statistically significant changes across all measurement timepoints. The analysis revealed significant improvements in both the WPI (*p* = 0.030) and FIQ scores (*p* = 0.044) across the treatment period (Figures 1, 2). While the SS-Score showed a positive trend (*p* = 0.108), it did not reach statistical significance, potentially due to the limited sample size (Figure 3).

Descriptive statistics demonstrated progressive reductions across all outcome measures. Median WPI scores decreased from 12 at baseline to 3 at the 30-day follow-up, while median FIQ scores improved from 65 to 6 over the same period. The consistency of therapeutic responses increased throughout treatment, as evidenced by narrowing confidence intervals in later sessions.

## 3 Discussion

This case series demonstrates that an integrative protocol combining PEMF and ozone therapy may induce clinically significant improvements across multiple statistical frameworks. At the individual level, all three patients exhibited statistically reliable changes in pain intensity (WPI) and functional impact (FIQ), while two of three showed reliable improvement in symptom severity (SS-Score). Complementing these findings, longitudinal analysis revealed statistically significant group-level improvements in both WPI (*p* = 0.030) and FIQ (*p* = 0.044) across the treatment period. The convergence of reliable individual changes and significant group-level improvements strongly suggests these benefits are unlikely to be due to chance or placebo effects alone.

The clinical improvements we observed may be explained by the complementary mechanisms of PEMF and ozone therapy. While PEMF is thought to influence cellular function, potentially reducing pain perception and inflammation, and improving sleep quality (5, 6), Ozone therapy likely contributes by modulating the immune system, reduces oxidative stress, and improves microcirculation and oxygen delivery to tissues, contributing to its analgesic and anti-inflammatory effects (7, 8). The combination likely produces a synergistic effect, addressing multiple pathophysiological aspects of FM.

A noteworthy secondary outcome was the patient-reported reduction in the use of conventional medications, particularly in Case 2, who was able to discontinue clonazepam and reduce her use of celecoxib. Notably, the maintenance of benefits at the 30-day follow-ups suggests the potential for this protocol to induce longer-lasting physiological changes. The progressive nature of these improvements, evidenced by the longitudinal analysis showing consistent score reductions across successive sessions, further supports the induction of cumulative physiological adaptations rather than transient symptomatic relief.

Despite the limitations inherent to its case series design, including the small sample size, lack of a control group, and potential placebo effects, the main strengths of this report include: the use of standardized, validated outcome measures (WPI, SS, FIQ); the application of both individual-level (RCI) and group-level (longitudinal Friedman tests) statistical analyses; the availability of multi-timepoint clinical data across five evaluation points, which were retrospectively compiled from clinical records with the inclusion of a 30-day follow-up period to assess durability; and the presentation of individual patient trajectories in supplementary timelines. The convergence of reliable individual changes and significant group-level improvements (WPI: *p* = 0.030; FIQ: *p* = 0.044) suggests these benefits are unlikely to be due to chance or placebo effects alone.

A significant limitation of this study is the absence of objective biomarkers. While the clinical improvements observed align with the proposed anti-inflammatory and antioxidant mechanisms of PEMF and ozone therapy, Inflammatory markers were not measured (e.g., CRP, IL-6), oxidative stress parameters (e.g., glutathione, malondialdehyde), or objective sleep metrics (e.g., polysomnography or actigraphy). The inclusion of such biomarkers in future randomized controlled trials would be invaluable to correlate clinical responses with physiological changes and to validate the hypothesized mechanisms of action.

Nevertheless, we recognize that randomized controlled trials with larger samples and sham-control conditions are urgently needed to validate these preliminary findings. Regarding prognosis, which is not typically staged in FM, the sustained improvement at the 30-day follow-up suggests a favorable short-term functional outlook for patients undergoing this integrative protocol.

In conclusion, this integrative, non-pharmacological protocol combining PEMF and ozone therapy appears to be a safe, effective, and well-tolerated option for managing FM. The significant improvements observed through both individual reliable change indices and group-level longitudinal analyses, coupled with reduced medication dependence, position this approach as a promising complementary strategy. Improvements sustained beyond the treatment period strongly support the initiation of larger, randomized controlled trials to rigorously validate its efficacy and explore its potential for inclusion in standardized clinical guidelines for FM management.

### 3.1 Patient perspective

All three patients reported favorable outcomes following the integrative protocol. Ke benefits emphasized included substantial improvement in their overall quality of life, citing a marked reduction in pain intensity, decreased fatigue, and better sleep quality. A particularly valued outcome was the reduced reliance on conventional medications, which was associated with feeling more mentally clear and experiencing fewer side effects. The non-invasive nature of the therapies and the absence of significant adverse events were also positively noted. Patients expressed that these improvements directly translated into a greater capacity to participate in daily social and occupational activities that were previously limited by their condition. These subjective reports of reduced pain, improved sleep, decreased fatigue, and enhanced daily functioning directly correlate with the objective improvements documented in the WPI, SS, and FIQ scores (Supplementary Figures 1–3), indicating a meaningful recovery of functional capacity and quality of life.

## Data Availability

The raw data supporting the conclusions of this article will be made available by the authors, without undue reservation.
