# Supplementary material for: Case Report: Integrative management of refractory fibromyalgia with pulsed electromagnetic fields and ozone therapy: a case series
Source: Front Med (Lausanne). 2026 Apr 15;13:1763506. doi: 10.3389/fmed.2026.1763506 (PMC13124560; doi:10.3389/fmed.2026.1763506)
Supplement: Supplementary file 2 [file Data_Sheet_2.pdf]

Supplementary Material

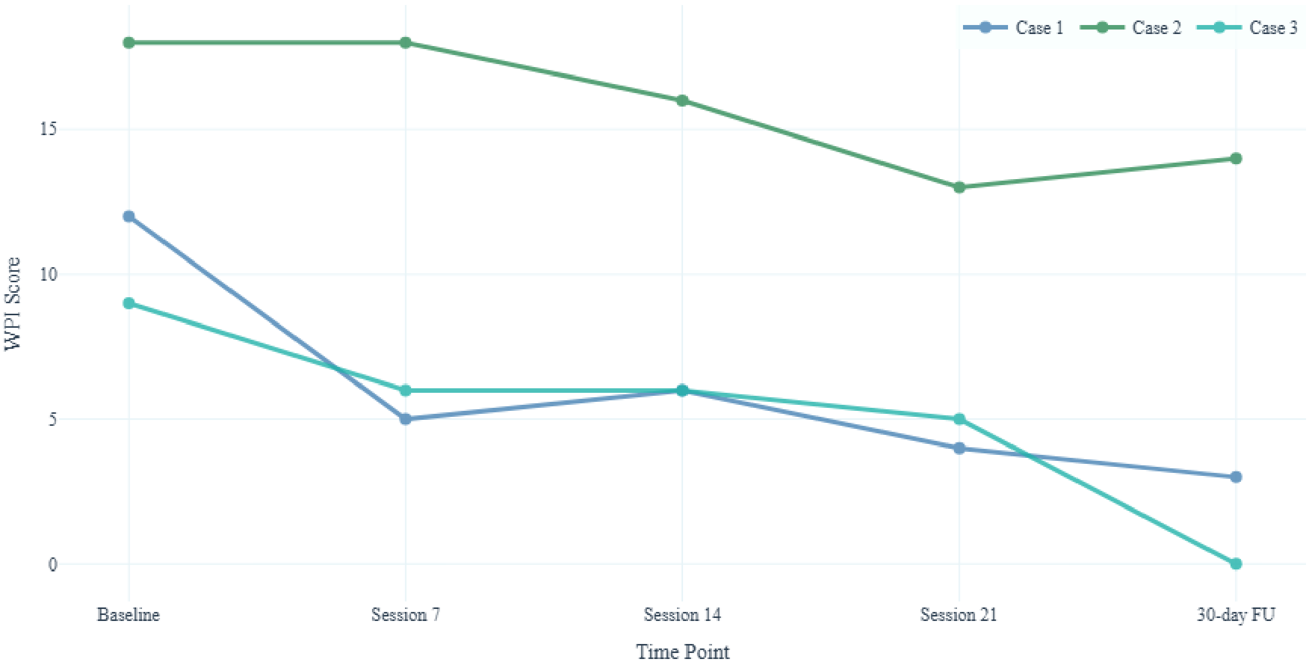

Supplementary Figure 1. Timeline of WPI scores demonstrates sustained reduction in pain areas across all three patients following the integrative PEMF and ozone therapy. Statistically reliable improvements (RCI: -5.84 to -6.57) were observed at the individual level. Benefits were maintained at 30-day follow-up (30 day FU), indicating durable effects beyond the active intervention period.

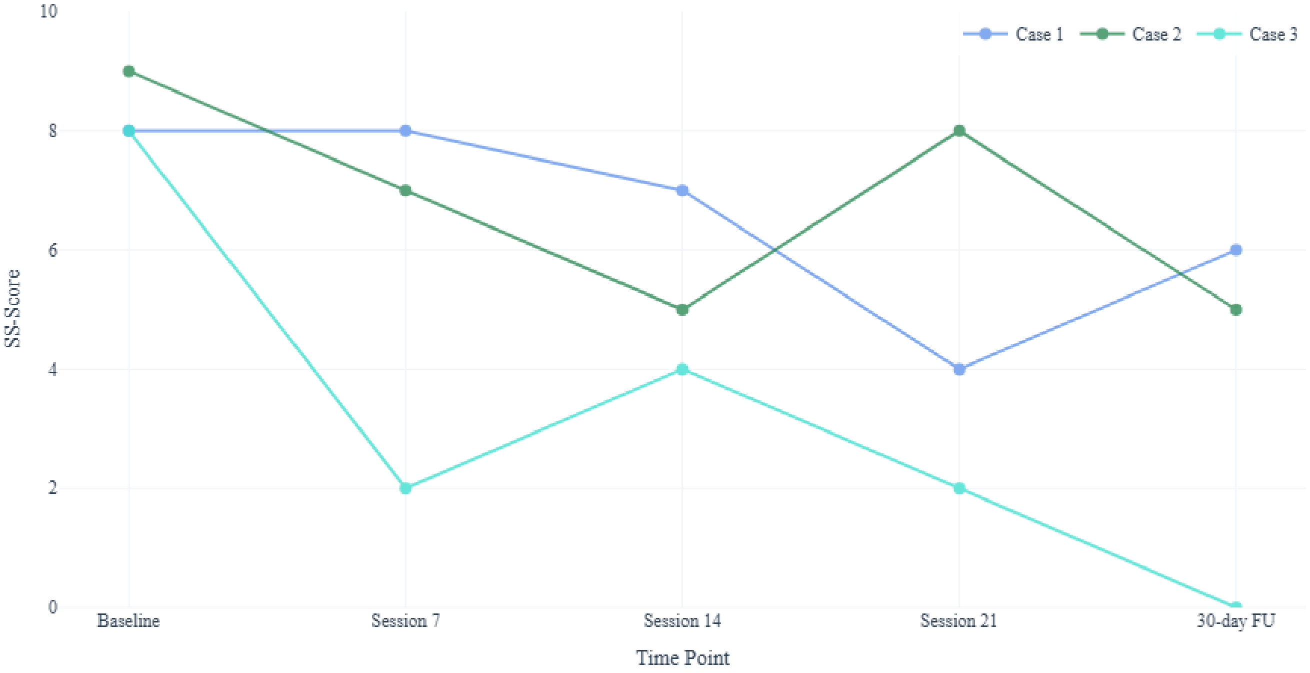

Supplementary Figure 2. Timeline of SS-Score scores show clinically significant improvement in

core FM symptoms for two patients (RCI: -4.08, -8.16), with stable maintenance at follow-up (30-day FU). Case 2 showed positive trend despite contextual stressors.

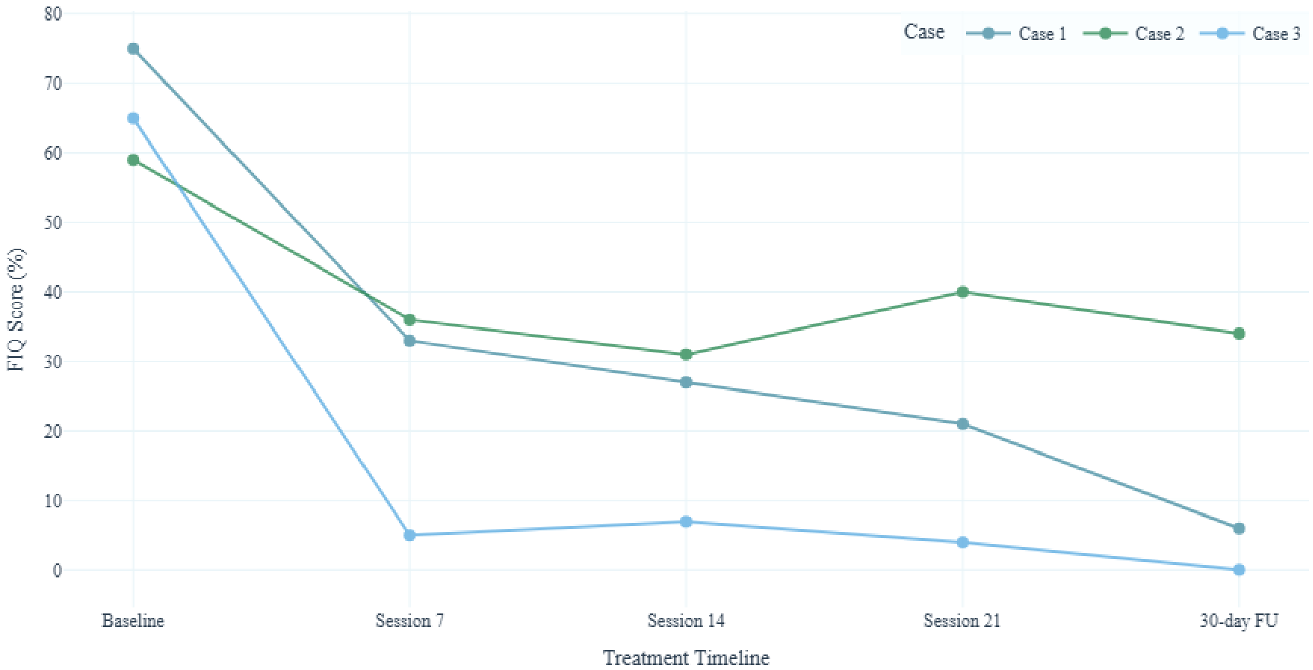

Supplementary Figure 3. Timeline of FIQ scores reveals substantial improvement in quality of life and functional impact. Reliable change indices (RCI: -7.98 to -9.01) confirmed individual treatment efficacy. Benefits persisted throughout the 30-day follow-up (30-day FU), suggesting lasting disease modification.

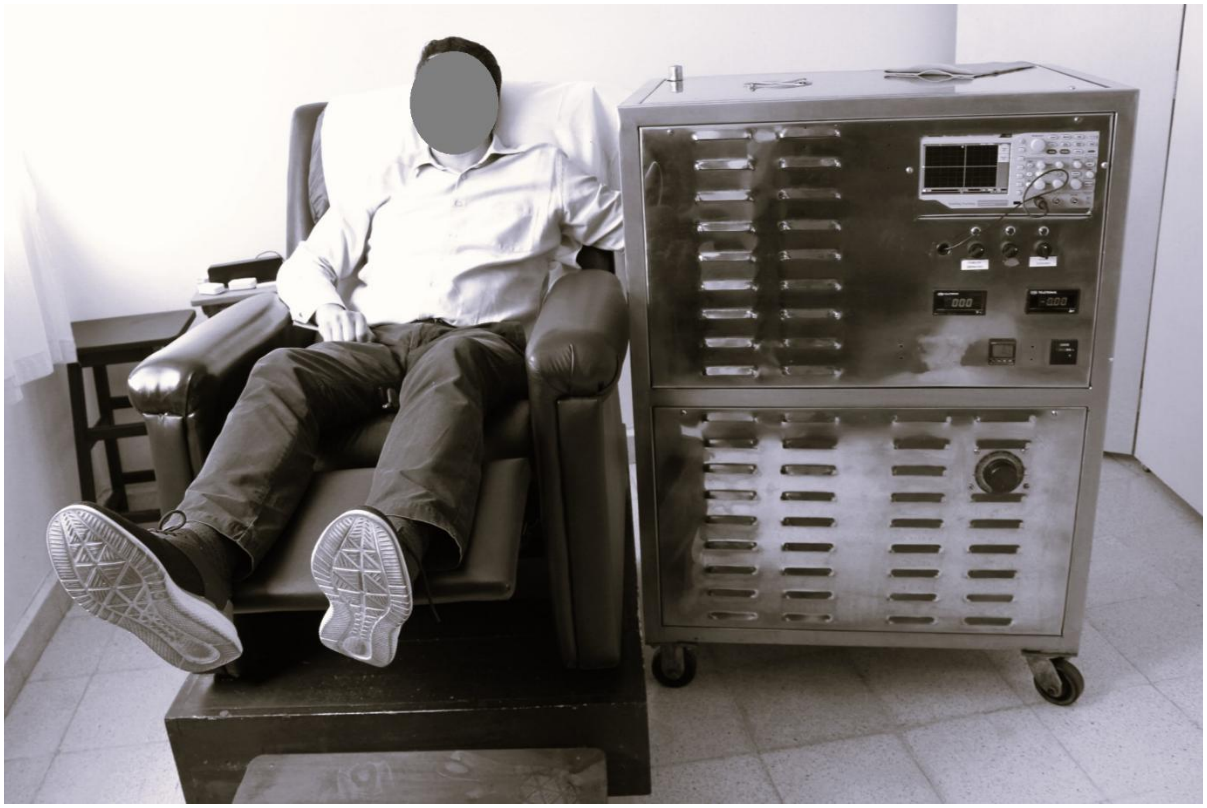

Supplementary Figure 4. EIMA pulsed electromagnetic field (PEMF) therapy system. The patient is shown in a resting position with the arm inserted into the lateral applicator for the administration of non-invasive systemic therapy.
